# Noninvasive Total Cholesterol Level Measurement Using an E-Nose System and Machine Learning on Exhaled Breath Samples

**DOI:** 10.1021/acssensors.4c02198

**Published:** 2024-11-22

**Authors:** Anna Paleczek, Justyna Grochala, Dominik Grochala, Jakub Słowik, Małgorzata Pihut, Jolanta E. Loster, Artur Rydosz

**Affiliations:** † AGH University of Krakow, Faculty of Computer Science Electronics and Telecommunications, Institute of Electronics, al. A. Mickiewicza 30, Krakow 30-059, Poland; ‡ Department of Prosthodontics and Orthodontics, Dental Institute, Faculty of Medicine, Jagiellonian University Medical College, ul. św. Anny 12, Kraków 31-008, Poland; § University Clinical Hospital in Opole, Institute of Medical Sciences, University of Opole, aleja Wincentego Witosa 26, Opole 46-020, Poland; ∥ Professor Loster’s Orthodontics, Private practice, Faculty of Medicine, Jagiellonian University Medical College, Bartłomieja Nowodworskiego 4, Krakow 30-433, Poland; ⊥ The University Hospital in Krakow, Laboratory of Functional and Virtual Medical 3D Imaging [3D-vFMi(maging)/3D-FM], Jakubowskiego 2 Street, Krakow 30-688, Poland

**Keywords:** E-nose system, exhaled breath analysis, gas sensors, LGBMRegressor, machine learning, noninvasive measurement, predictive modeling, total cholesterol level

## Abstract

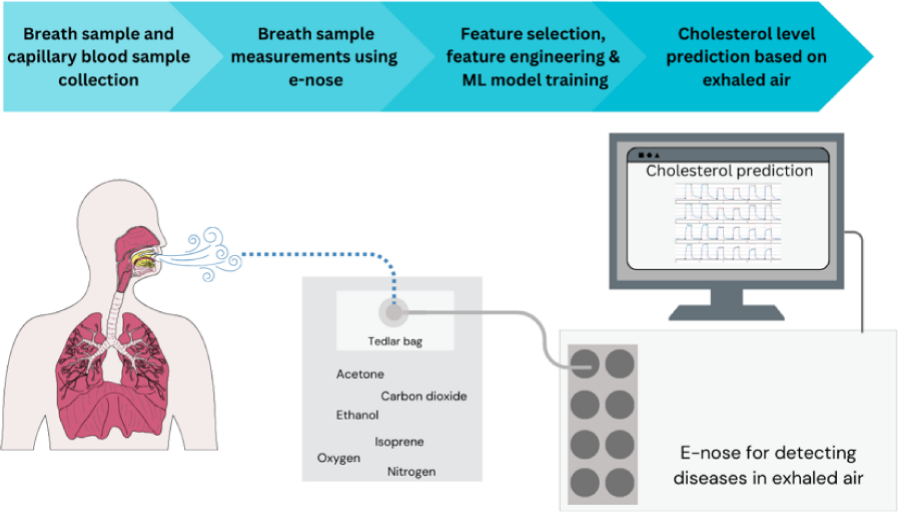

In this paper, the first e-nose system coupled with machine learning algorithm for noninvasive measurement of total cholesterol level based on exhaled air sample was proposed. The study was conducted with the participation of 151 people, from whom a breath sample was collected, and the level of total cholesterol was measured. The breath sample was examined using e-nose and gas sensors, such as TGS1820, TGS2620, TGS2600, MQ3, Semeatech 7e4 NO2 and 7e4 H2S, SGX_NO2, SGX_H2S, K33, AL-03P, and AL-03S. The LGBMRegressor algorithm was used to predict cholesterol level based on the breath sample. Machine learning algorithms were developed for the entire measurement range and for the norm range ≤200 mg/dL achieving MAPE 13.7% and 8%, respectively. The results show that it is possible to develop a noninvasive device to measure total cholesterol level from breath.

## Volatile Organic Compounds

**Figure 1:**
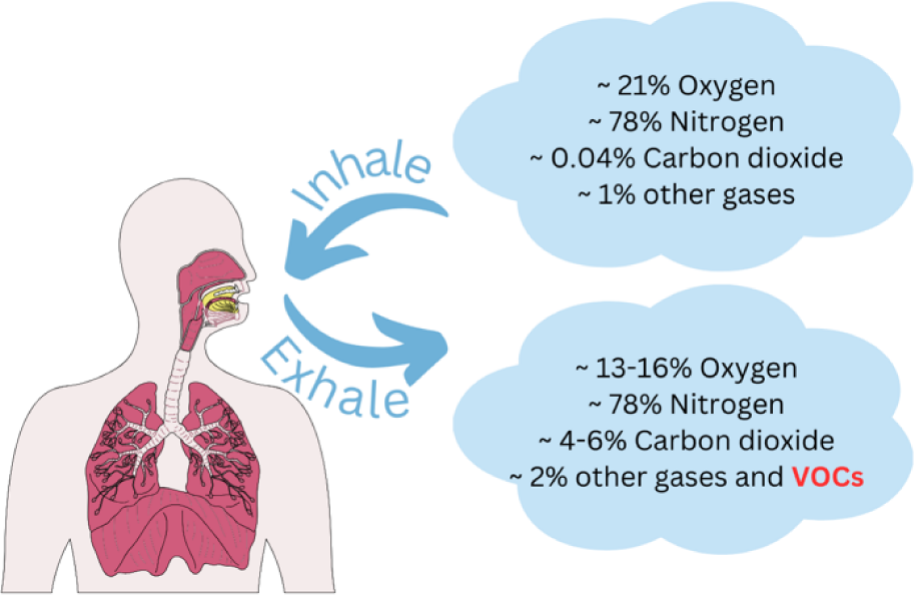
Composition of inhaled and exhaled air.

In recent times, researchers have been working to develop noninvasive methods for measuring and monitoring health parameters, such as blood glucose levels,^1−4^ detection of FeNO for asthma and other respiratory diseases,^5,6^ SIBO (small intestine bacterial overgrowth), or various types of cancers.^7−9^ One possibility is to monitor exhaled breath and the volatile organic compounds (VOCs) contained in it. Human breath (Figure 1) consists mainly of nitrogen (78%–79%), oxygen (13%–16%), and carbon dioxide (4%).^10^ The rest of the parts are mostly VOCs. Currently, over 3,000 different VOCs have been identified in breath.^11^ Some of them may be of endogenous origin and may be biomarkers of various diseases or conditions of the human body and come from metabolic processes occurring in the body. Exogenous VOCs are the result of external factors, such as smoking, air pollution, or drug metabolism.^12^ The relative humidity of human breath is 89%–97%.^13^

## Clinical Importance of Monitoring Blood Cholesterol Levels

Cholesterol performs an essential role in human metabolism and permits homeostatic regulation. It is a crucial component of every cell membrane.^14^ As a steroid hormones’ precursor, cholesterol is responsible for various immune, development and reproductive processes as well as mineral metabolism.^15^ Despite this biological significance, hypercholesterolemia contributes to the pathogenesis of cardiovascular diseases (CVDs)—a leading cause of death worldwide. According to the WHO 17.9 million people die of CVDs every year.^16^ Cholesterol can accumulate in the walls of arteries and form atheromatous plaques. After long asymptomatic period, plaque can rupture causing intravascular coagulation and ischemia. This phenomenon occurs particularly within the coronary, cerebral, and peripheral circulation, leading respectively to myocardial infarction, stroke, and limb ischemia. It is estimated that up to 90% of CVDs could be avoided by modifying risk factors.^17^ Hypercholesterolemia is one of the most important modifiable risk factors for CVDs, so regular assessment of cholesterol levels and early implementation of appropriate treatment are valuable for patients. Although the clinical use of total cholesterol (TC) in relation to the LDL-cholesterol (LDL-C) is very limited, a linear correlation of TC levels with cardiovascular risk has been demonstrated.^18^

## The Relationship Between Blood Cholesterol and VOCs

**Figure 2:**
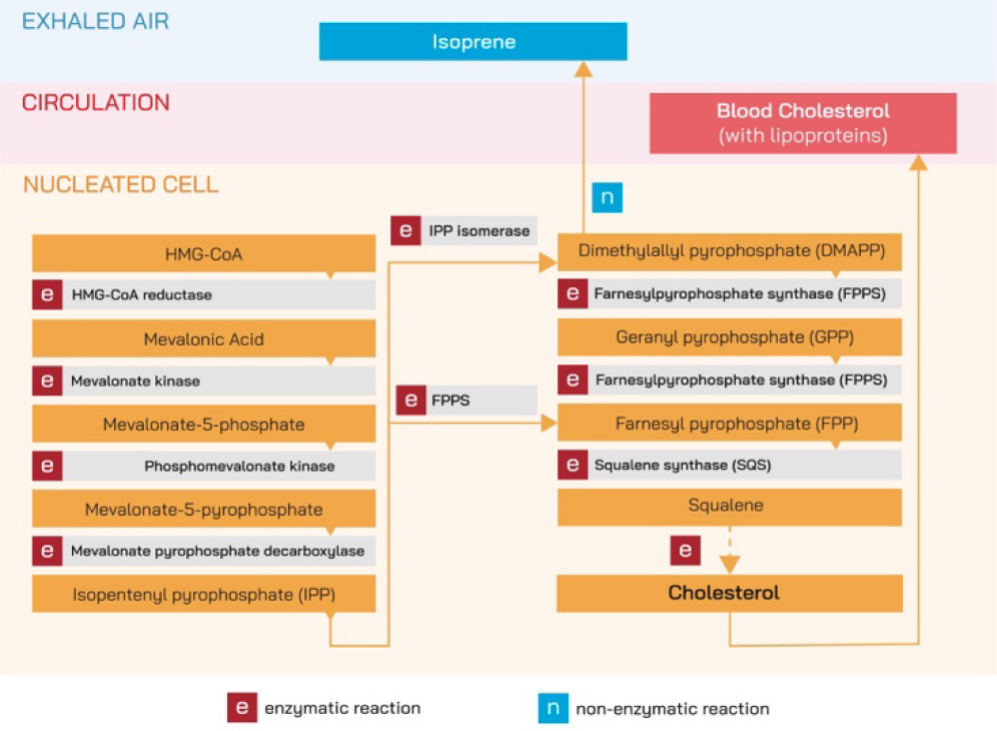
Metabolic pathway of cholesterol and its relationship to isoprene in breath.

It is presumed that isoprene is formed during cholesterol biosynthesis in nucleated cells by nonenzymatic conversion of DMAPP. Thereafter, it enters the alveoli via the vascular system and is excreted with exhaled air. The metabolic pathway of cholesterol and its relationship to isoprene in breath^19−22^ is shown in Figure 2.

## Gas Sensing Methods

VOCs in breath occur in concentrations of parts per million (ppm), parts per billion (ppb), or even parts per trillion (ppt); therefore, determining their concentration in exhaled air is difficult using the commercially available gas sensors. There are reference methods, such as gas chromatography coupled with mass spectrometry,^23,24^ selected ion flow-tube mass spectrometry,^25^ proton-transfer-reaction time-of-flight mass spectrometry,^26^ which allow for the separation of gas mixtures into components and their quantitative analysis. The operation of such devices is complicated, they require special storage or long-term start-up procedures, and they are very expensive. Therefore, gas sensors for detecting low concentrations of compounds in gas mixtures have been widely developed. Because sensors can detect multiple substances and exhaled air contains numerous volatile organic compounds, employing a matrix of gas sensors and machine learning algorithms is essential to increase the sensitivity and selectivity of the e-nose systems.

## Breath Sampling Methods

Most often, breath is collected in bags specially designed for this purpose, which maintain the initial concentration of compounds contained in the gas mixture for up to several days.^27−29^ Such bags include Tedlar Bag, FlexFoil PLUS.^30^ It is also possible to supply exhaled air directly to the device.^31,32^ Currently, there is no standardized method for storing and collecting breath samples, which leads to problems with reproducing studies and comparing results with those of other researchers.

## Related Works

In the related literature, the study of exhaled isoprene and its relationship with cholesterol concentration is often mentioned, but no studies using e-nose to estimate cholesterol from exhaled breath have been presented yet. Gouma et al. proposed a selective nanosensor for exhaled breath analysis, which can be used for noninvasive monitoring of cholesterol levels. They developed sensor arrays for measuring isoprene, carbon dioxide and ammonia gas, however the sensor was tested only on synthetic gases that were composed to mimic human exhaled air.^33^ Similar research was conducted by Güntner et al., who developed a Ti-doped ZnO sensor for selective sensing of isoprene for breath diagnosis. This sensor showed a significantly higher response to isoprene than to acetone, ammonia, or ethanol at 90% RH, which is the observed RH of human breath. In this case the authors also tested the sensor only on synthetic gas mixtures.^34^

This paper introduces the first e-nose system combined with a machine learning algorithm for noninvasive measurement of total cholesterol levels using exhaled air samples. The study involved 151 participants from whom a breath sample was collected, and the level of total cholesterol was measured.

## Experimental Section

### Information About the Study Involving Human Participants

In collaboration with the Department of Prosthodontics and Orthodontics at the Dental Institute, Faculty of Medicine, Jagiellonian University Medical College, Krakow, Poland, tests were conducted on breath samples and capillary blood samples collected from 151 individuals (Jagiellonian University bioethical committee approval KBET: 1072.6120.40.2023). The study included patients over the age of 45 to identify those at risk of developing features of metabolic syndrome.

### Patients’ Information

Each of the 151 participants completed a questionnaire that included questions about gender, weight, height, age, medications taken, past and current illnesses, and well-being related to the use of dentures and dental cavities. 92 women and 59 men participated in the study. Descriptive statistics of the sample population including data on participants’ age, height, weight, and BMI are included in Table 1.

**Table 1:** Descriptive Statistics of the Sample Population

Parameter	Mean	Standard Deviation
Age	67	9.3
Weight	77 [kg]	15.5 [kg]
Height	166 [cm]	9.4 [cm]
BMI	27.7 [kg/m^2^]	4.11 [kg/m^2^]

### Capillary Blood Tests

Participants in the study had their capillary blood samples analyzed by a physician using devices that measure parameters via the strip technique, such as

- Glucose (Accu-Chek Instant, Roche Diabetes Care GmbH, Sandhofer Strasse 116, 68305 Mannheim; www.roche.com.).
- Uric acid (PEMPA 3in1 device, General Life Biotechnology Co., Ltd. 5F., No. 240, Shinshu Rd., Shin Juang Dist., New Taipei City 242, Taiwan; www.BeneCheck.com.tw.).
- Cholesterol (PEMPA 3in1 device, General Life Biotechnology Co., Ltd. 5F., No. 240, Shinshu Rd., Shin Juang Dist., New Taipei City 242, Taiwan; www.BeneCheck.com.tw.).
- Triglycerides (Accutrend Plus, Accutrend Glucose, Roche Diagnostics GmbH, Sandhofer Strasse 116, 68305 Mannheim; www.roche.com).

Descriptive statistics of blood test parameters, including data on measured values of glucose, uric acid, cholesterol, and triglycerides from capillary blood of the participants, are included in Table 2.

**Table 2:** Descriptive Statistics of Blood Test Parameters

Parameter	Mean	Standard Deviation
Glucose	110.5 [mg/dL]	31.32 [mg/dL]
Uric acid	5.66 [mg/dL]	1.49 [mg/dL]
Cholesterol	174.33 [mg/dL]	39.02 [mg/dL]
Triglycerides	124.5 [mg/dL]	84.53 [mg/dL]

### Cholesterol Levels Distribution

**Figure 3:**
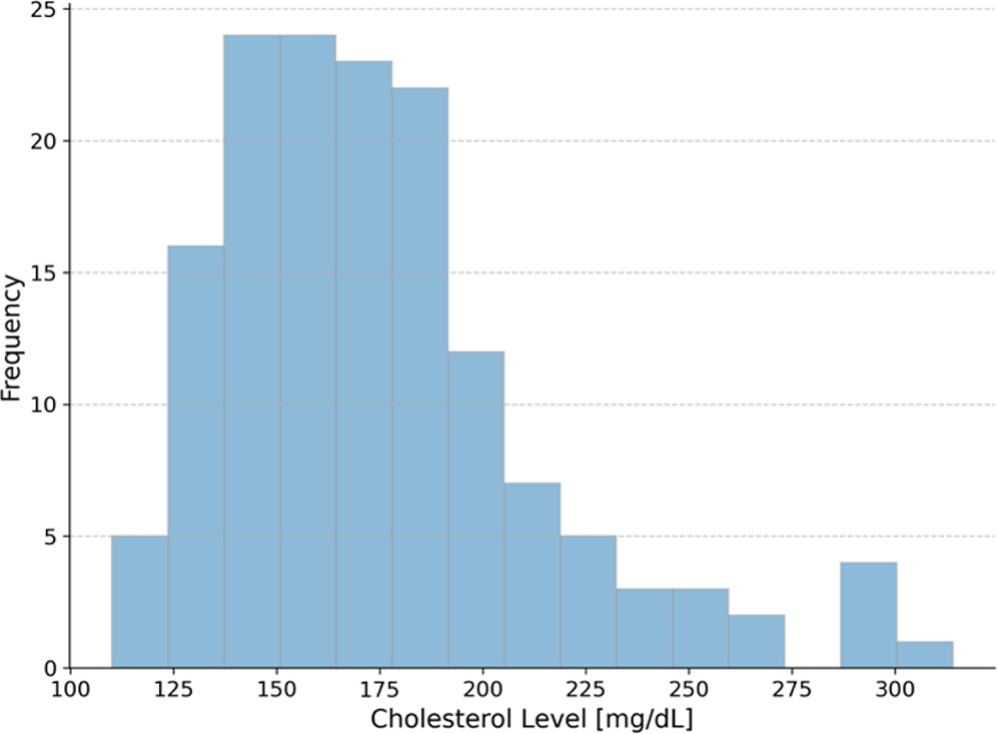
Distribution of cholesterol levels among the study participants.

In this paper, we focus on predicting cholesterol levels based on exhaled air measurements. The PEMPA 3-in-1 device allows cholesterol to be measured from fresh capillary blood in the range of 100–400 mg/dL (2.59–10.35 mmol/L). With this test, the norm is a result of ≤200 mg/dL (5.17 mmol/L).^35^ The distribution of cholesterol values measured in the study participants is presented in Figure 3.

### Breath Tests

**Figure 4:**
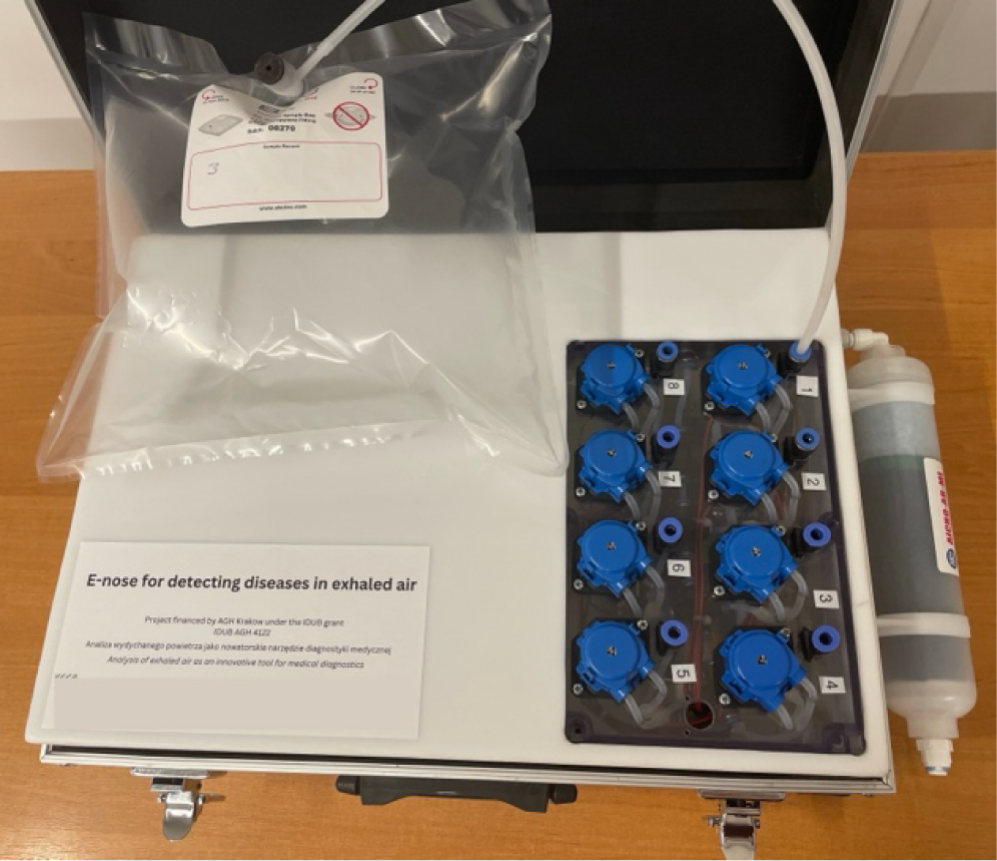
E-nose system used during measurements.

Breath samples were collected in Tedlar bags and analyzed using an electronic nose (e-nose) twice. Tedlar bags are specialized bags for collecting and storing breath samples. Their advantage is maintaining high concentrations of the collected substances, which allows the bags to be transported to the external laboratory and to cooperate with remote research centers or hospitals.^28,36−38^ However, the e-nose system that we propose is portable and allows for quick testing of the sample in a hospital or medical center (Figure 4).

### E-Nose System

The e-nose comprised a system for pumping air from the bags and a set of sensors, including TGS1820, TGS2620, and TGS2600 (Figaro Engineering Inc., Mino, Osaka, Japan), MQ3 (Winsen, ZhengZhou, HeNan, China), 7e4 NO2, 7e4 H2S (SemeaTech, Los Angeles, USA and Shanghai, China), SGX_NO2, SGX_H2S (SGX SENSORTECH, Switzerland), K33 (Senseair, Delsbo, Sweden), and AL-03P, AL-03S (MGK SENSOR Co., Ltd., Saitama, Japan).

### Sensors’ Responses

**Figure 5:**
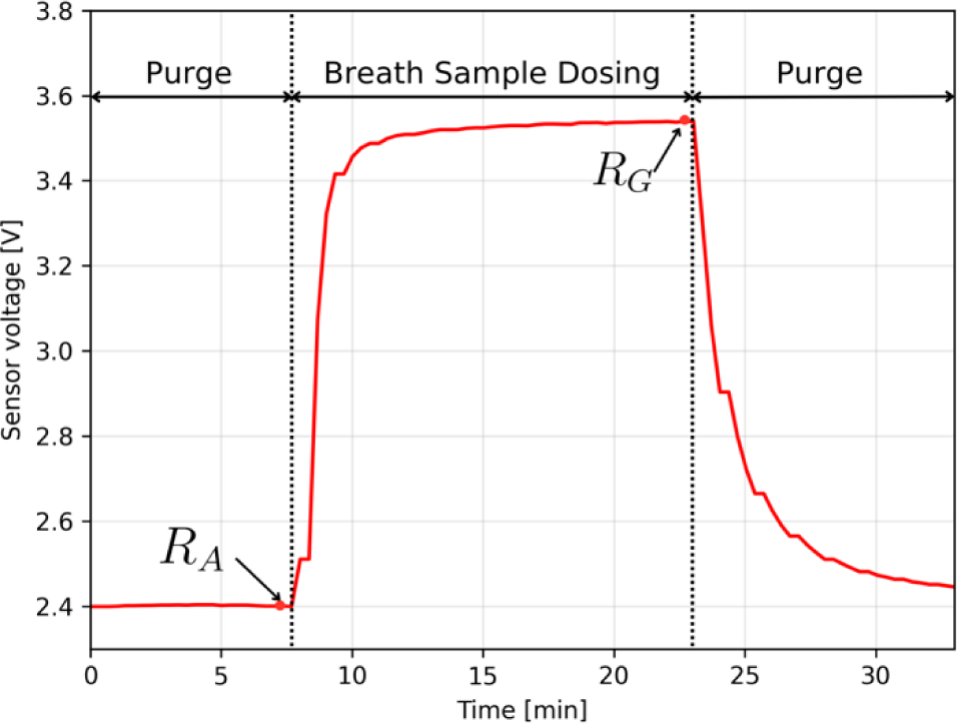
Stages of the breath sample measurement using the developed e-nose system.

As part of the study, the breath sample collected from each patient in a Tedlar bag was measured twice using the prepared e-nose system. The time of rinsing with ambient air collected through the filter was 10 min between subsequent measurements, and the time of air injection from the bag was 15 min. For each measurement, the *R*_A_ (sensor response to purge gas) and *R*_G_ (sensor response to breath sample) values were determined (as shown in Figure 5) and the responses of the *S* and *S*_1_ sensors were calculated (eqs 1 and 2).
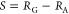
1
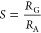
2

Gas sensor data typically consist of electrical values affected by measurement errors, noise, or drift^39,40^ due to changes in sensor layer properties. These quality issues can impact model training and performance, so researchers use signal processing techniques like filtering^41,42^ and baseline normalization,^39,43,44^ to prepare the data for next steps in the processing pipeline. In our solution, we used a mean filter^41^ to reduce noise, calculating an average of 10 samples, while calculating the sensor response (eqs 1 and 2), which takes into account the baseline value (in the sensor response in the purge stage), allows to minimize the influence of drift. Additionally, before testing using human breaths, the sensors were tested on synthetic mixtures and results were published in our previous papers.^43,45^

### Outliers Handling

Based on the sensor responses, four outliers were removed for the K33 (CO_2_ sensor) and AL-03P (ethanol sensor) sensors. Measurements were removed where the K33 sensor measured a CO_2_ value lower than 2%, which means that the breath sample was incorrectly collected, and measurements where the AL-03P sensor indicated a response indicating the presence of ethanol in exhaled air, which could come from the mouthwash.

### Train Test Split

The data set was divided into training and test sets in a ratio of 90:10 so that both measured values of the breath sample of one patient were located in only one of the sets. The training set included breath sample measurements collected from 136 patients, and the test set included 15 patients. This means that when two measurements from each patient were used, the training and test sets included 272 and 30 samples, respectively.

### Machine Learning Algorithms

The aim of the study was to develop an algorithm that would allow prediction of cholesterol concentration in blood using e-nose and breath sample. The *R*_G_, *S*, and *S*_1_ data from sensors available in e-nose and BMI were taken as features. For this purpose, machine learning algorithms were used for the regression problem. The study tested machine learning algorithms: linear regression, lasso regression, ridge regression, random forest, LGBM regressor, XGB regressor, CatBoost regressor, KNN regressor, and neural networks. The results for all algorithms were compared (Table 3) and the best results were obtained using LGBM regressor. For each algorithm, the hyperparameter space for searching was determined. The best hyperparameters were determined using the RandomSearchCV^46,47^ method from the scikit-learn library (30 splits, negative mean absolute error optimization)

**Table 3:** Comparison of Machine Learning Algorithm Performance (Measured as Mean Absolute Error) in Total Cholesterol Level Prediction (Norm Range)

Algorithm	Mean absolute error
Linear Regression	17.02
Lasso Regression	20.82
Ridge Regression	16.43
Random Forest	17.11
LightGBM Regressor	12.94
XGBoost Regressor	19.41
CatBoost Regressor	16.84
KNN Regressor	19.11

### LightGBM Regressor Model

LightGBM is a gradient boosting framework that employs tree-based learning algorithms designed for distribution and efficiency. It offers several key benefits including faster training speed, higher efficiency, and lower memory usage. Additionally, it provides better accuracy and supports parallel, distributed, and GPU-based learning, making it capable of handling large-scale data sets effectively.^48^ Linear regression models, lasso, and ridge, assume linear relationships between variables, which is a major limitation in the case of sensors’ data processing. LGBM, like random forest, CatBoost, and XGBRegressor, is a tree model that can better handle nonlinear relationships in the data.^49^ LightGBM handles large numbers of features very well, which can lead to more accurate predictions, even when other models may struggle to maintain performance. LightGBM has parameters that allow for overfitting control (e.g., max_depth, num_leaves, and feature_fraction). This makes it easy to tune to generalize well to the data, which is an advantage oversimpler models, such as linear regression, that have limited overfitting control. Additionally, LGBMRegressor has built-in function for feature importance calculation and analysis.^50^

### Metrics

The following metrics were used to evaluate the effectiveness of regression algorithms: mean absolute error (MAE), root-mean-square error (RMSE), mean absolute percentage error (MAPE), and *R*^2^ coefficient.

## Results and Discussion

### Cholesterol Level Distribution Analysis

The distribution of measured cholesterol levels in the patients is previously shown in Figure 3. The analysis of the histogram and the values of mean (174.33), median (166.0), and calculated skewness index (1.18) shows that the distribution of cholesterol level among the patients participating in the study is right-skewed (the skewness coefficient is greater than 0 and the mean is greater than the median). Twenty-six patients had a score above 200 mg/dL (norm result) and only 7 above 260 mg/dL.

Considering the aforementioned problem, we decided to train two separate algorithms.

Prediction of cholesterol level in the entire range.
Prediction of cholesterol level within the norm (≤200 mg/dL).

Additionally, the predicted value logarithm technique was used to limit the influence of skewness.^51^ For prediction over the full range, we obtained better results using only one measurement for each patient. The results for both cases are compared in Table 4.

**Table 4:** Comparison of Metrics for the Entire Range and Norm Range Prediction using LGBM Regressor

Metric	Entire range	Norm range
MAE	21.2	12.9
RMSE	26.4	15.8
*R*^2^	0.22	0.52
MAPE	13.7%	8%

### Prediction of Cholesterol Level in the Entire Range

**Figure 6:**
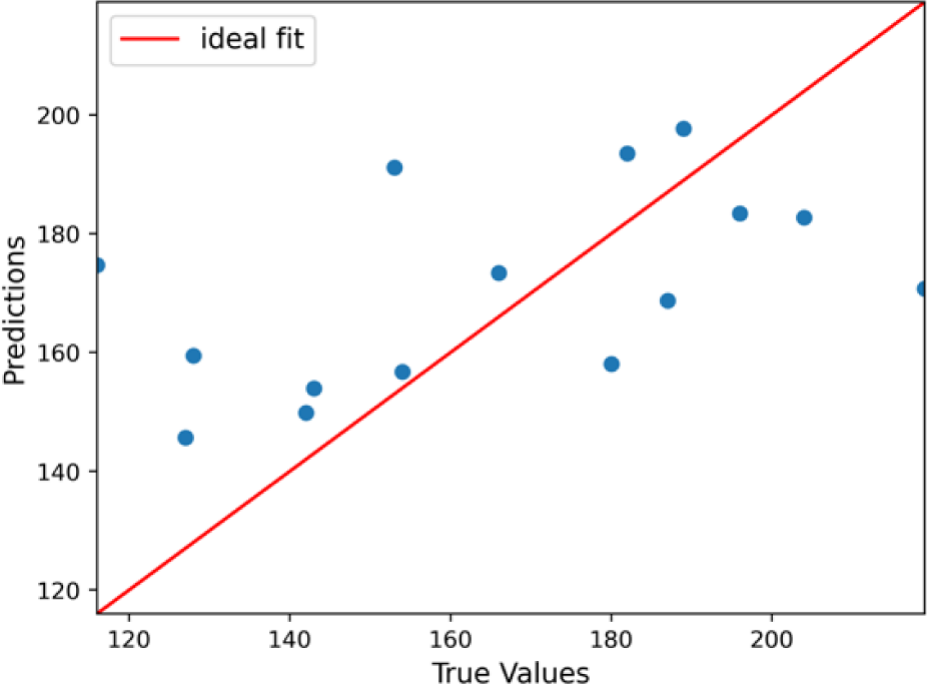
Results of prediction of the total cholesterol level in the entire range.

On average, the predicted cholesterol levels deviate from the actual values by about 21.22 mg/dL. The *R*-squared value indicates how well the model explains the variance in the target variable. An *R*^2^ of 0.224 means that the model explains about 22.4% of the variance in cholesterol levels, which is relatively low. A MAPE of 13.73% means that, on average, the model’s predictions are about 13.73% off from the actual cholesterol levels. A comparison of the values predicted by the machine learning algorithm based on breath sample testing and the values measured using the test strip and capillary blood is shown in Figure 6.

### Prediction of Cholesterol Level within the Norm

**Figure 7:**
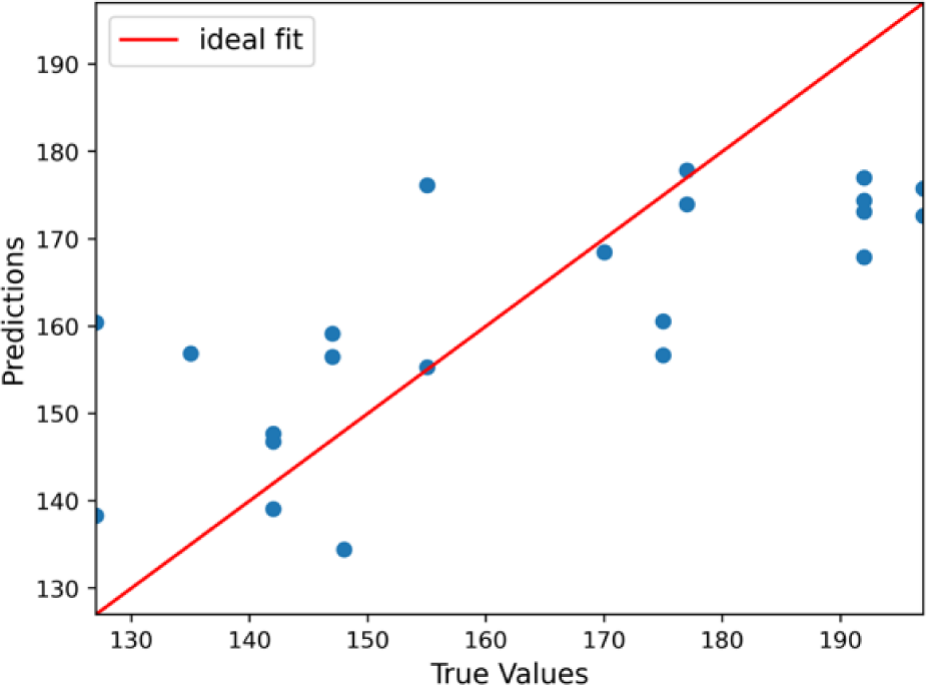
Results of prediction of the total cholesterol level in the norm range.

The performance metrics obtained from the prediction model for total cholesterol levels in the norm range based on exhaled air are indicative of a quite successful model. On average, the predicted cholesterol levels deviate from the actual values by approximately 12.94 mg/dL. This RMSE value indicates that the typical prediction error is around 15.79 mg/dL, providing a more substantial penalty for larger errors. The *R*-squared (*R*^2^) value of 0.522 signifies that the model explains about 52.2% of the variance in the cholesterol levels, which is moderately good but also highlights that there is room for improvement. Additionally, the model’s predictions are, on average, within 7.99% of the actual values. These results suggest that while the model has a reasonable predictive capability, further refinement, additional features, and additional data could enhance its accuracy and reliability. A comparison of the values predicted (in norm range) by the LGBMRegressor algorithm based on breath sample testing and the values measured using the test strip and capillary blood is shown in Figure 7.

**Figure 8:**
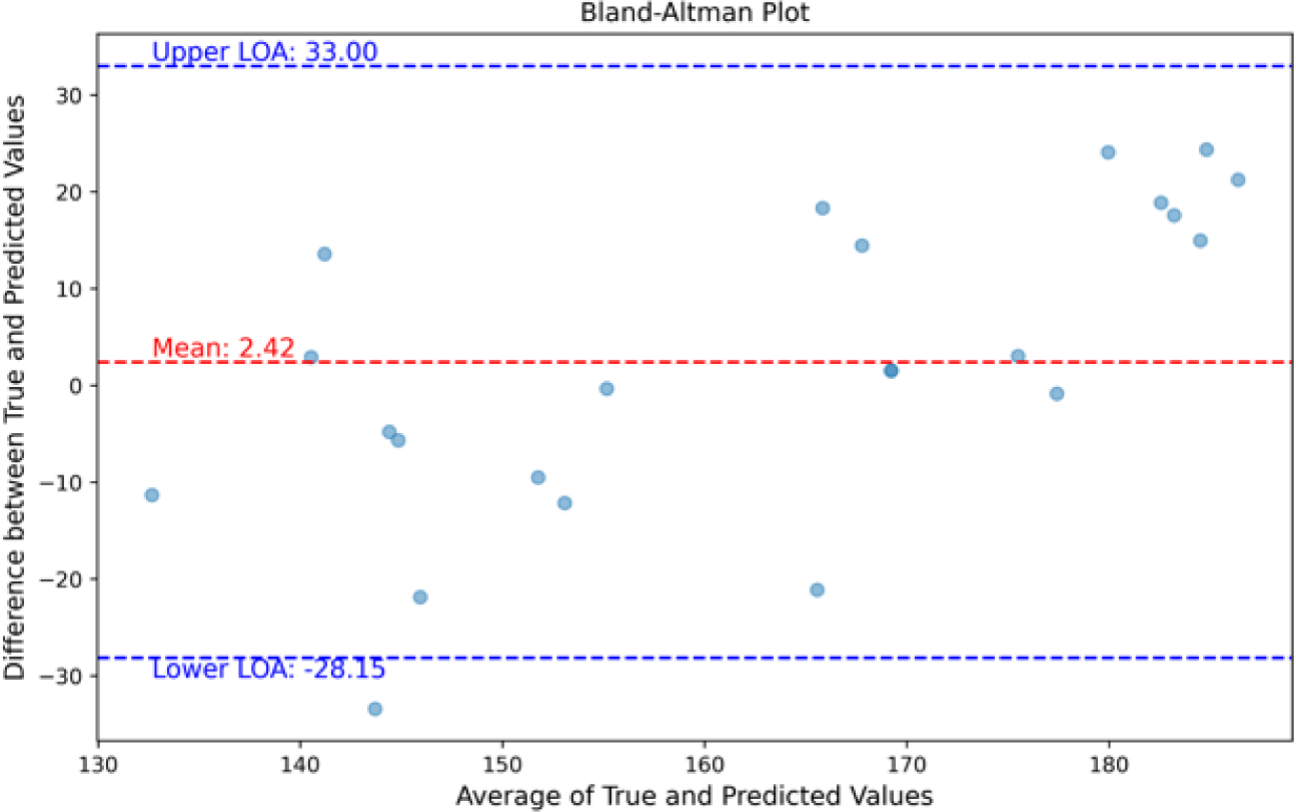
Bland-Altman plot of predictions of total cholesterol level in the norm range.

The Bland– Altman plot analysis provides further insight into the agreement between the predicted and actual total cholesterol levels. The mean difference (or bias) between the predictions and the actual measurements is 2.42 mg/dL. This small mean difference indicates that, on average, the model slightly overestimates the cholesterol levels by 2.42 mg/dL. The limits of agreement (LOA) are defined as the mean difference plus and minus 1.96 times the standard deviation of the differences. The upper LOA is 33.00 mg/dL, and the lower LOA is −28.15 mg/dL. This range suggests that 95% of the differences between the predicted and actual cholesterol levels fall within this interval. The Bland-Altman plot is illustrated in Figure 8.

### Features Importance

Analysis of the most important features showed that the most important for prediction were the responses of the TGS1820, AL-03P, TGS2620, and MQ3 sensors. These are mainly sensors for acetone, ethanol, and VOCs. Isoprene, which is most often observed as a cholesterol biomarker in breath, is also a volatile organic compound and can be detected by semiconductor sensors, such as TGS1820 or TGS2620. Gas sensors, especially those based on metal oxides (e.g., SnO_2_), operate on the principle of electrical conductivity change in the presence of volatile organic compounds. Isoprene, being a VOC, can cause a change in conductivity similar to that of acetone or ethanol. Due to the cross-selectivity of sensors and the large number of VOCs in exhaled air, it is necessary to use a gas sensor matrix and machine learning algorithms.

## Conclusions

In this paper, we proposed the first e-nose for prediction of total cholesterol concentration in blood based on the exhaled breath analysis. Machine learning algorithms were developed for the entire measurement range and for the norm range ≤200 mg/dL achieving MAPE 13.7% and 8%, respectively. These are the first results allowing further development of the solution and achieving better results. One of the limitations of our study was that only 151 people participated in the study, which is a good introduction to research, while a larger population would improve the results. Total cholesterol level values observed in patients have a right skewed distribution and a small number of people achieved results above the norm, which was difficult for the model to generalize; however, the results in the norm range, where the number of patients was higher, show that such prediction is possible, and it is possible to achieve smaller errors with a larger population. One of the disadvantages of our study is that as a method of determining total cholesterol level in blood, we adopted a portable device for a capillary blood test strip and not measurements from venous blood performed in a professional laboratory with venous blood samples. Measurements with such a device are also burdened with measurement errors. Studies and reports show that the mean absolute relative difference of the five cholesterol self-tests ranged from 6 ± 5% (Accutrend Plus) to 20 ± 12% (Mylan Mytest).^52,53^ Our study included people who fasted before the test and those who fasted after a meal. Studies show that there are no clinically significant differences in the level of total cholesterol in the blood after fasting and after a meal.^54^ Our method copes with both cases.

Human breath is composed of many compounds that reflect the state of the body but also affect the response of sensors and the prediction of algorithms. Factors that can distort the results include external air pollution,^12,55^ smoking, drinking, or eating immediately before the test. In addition, medications taken or other co-occurring diseases also have an impact. Often, when patients have metabolic syndrome^56^ (as was the case in our studies), a simultaneous increase in blood parameters such as cholesterol, blood glucose level or triglycerides is observed. Therefore, it is important to collect additional data about patients, as well as to determine the patient’s behavior before the test, just as is done with standard blood tests.

The next stages of the study development are the development of a portable device that would allow for broader screening of patients in various medical centers and comparison of results with total cholesterol determined in venous blood. One of the possibilities is also the study of additional parameters such as LDL-C and HDL-C levels and an attempt to predict them based on breathing. In summary, our study and the developed e-nose with machine learning algorithms provide a good basis for further research on a larger population and the development of a portable device for noninvasive prediction of total cholesterol, HDL-C and LDL-C levels based on a breath sample.

## Abbreviations

CVDs: cardiovascular diseases
HDL-C: HDL-cholesterol
LDL-C: LDL-cholesterol
MAE: mean absolute error
MAPE: mean absolute percentage error
ppb: parts per billion
ppm: parts per million
ppt: parts per trillion
RH: relative humidity
RMSE: root mean square error
TC: total cholesterol
VOCs: volatile organic compounds
